# Children’s Centers Study Kids and Chemicals

**DOI:** 10.1289/ehp.113-a664

**Published:** 2005-10

**Authors:** Melissa Lee Phillips

Many studies in recent years have documented that whether they’re used to spray in the kitchen or spray in the field, pesticides have a way of getting into almost all human environments. Pesticide exposure isn’t a great idea for adults, but it poses a particular concern in regards to children. These smallest humans, who spend a lot of time close to the floor and with their hands in their mouths, can encounter much higher doses relative to their body weights. And because they are still growing and developing, children are often more vulnerable to adverse effects of these and other environmental exposures. Likewise, the developing fetus may be especially vulnerable to the effects of pesticide exposure *in utero*.

In 1998, the NIEHS joined with the U.S. Environmental Protection Agency (EPA) to create eight centers across the country where scientists study environmental influences on children’s health. Today there are 11 centers. Several of these centers, including those at Columbia University and Mount Sinai School of Medicine in New York City, the University of California (UC), Berkeley, and the University of Washington (UW) in Seattle, have focused their efforts on pesticide exposures—how they occur, and the effects they cause *in utero* and during early childhood. These centers have also studied exposures to other environmental toxicants such as polycyclic aromatic hydrocarbons (PAHs), polychlorinated biphenyls (PCBs), and environmental tobacco smoke.

These studies are showing that children in certain communities have elevated exposures to toxicants early in their development and that some of these exposures can lead to slightly stunted fetal growth, shorter gestation, and suboptimal neurodevelopment, as well as to predisposition to diseases such as asthma. Additional studies are showing that the potential for damage from these chemical exposures may be affected by genetic susceptibility of both the child and the mother. Thus, interactions between genes, the environment, and the timing of exposure can all contribute to a later susceptibility to develop diseases and disorders.

## Columbia University

“Early-life exposures, even occurring in the womb, appear to be important determinants of that child’s respiratory health and development later on,” says Frederica Perera, director of the center at Columbia University and a professor of public health. “We have enormous opportunities to prevent these diseases and conditions.” At Columbia University, researchers have set up a cohort study to analyze exposure to pesticides and PAHs during pregnancy and very early childhood, a time of susceptibility that Perera says has not been adequately studied in the past.

Since 1998, nearly 700 pregnant Dominican and black women from Washington Heights, Harlem, and the South Bronx have enrolled in the study. Researchers take urine samples from mothers during pregnancy and blood samples from their babies at birth, sample ambient air in the mothers’ environment during pregnancy, and administer questionnaires and biomarker assessments repeatedly over the child’s early years.

Perera and her colleagues found that all the women in their cohort—and, therefore, their developing babies—were exposed to PAHs from vehicle exhaust and to at least one neurotoxic pesticide during pregnancy. In the February 2003 issue of *EHP* they reported finding that high PAH levels in a mother’s air samples correlated with having a smaller baby at birth. In papers published in the *American Journal of Respiratory and Critical Care Medicine* between 2000 and 2002, the team further reported that high prenatal exposure to certain PAHs was related to an increased likelihood that children would show asthma precursor symptoms and allergic responses to cockroach, mouse, and dust mite allergens at 2 years of age.

“We also see evidence that [PAH] exposures can influence cancer risk,” Perera says. Prenatal exposure to PAHs was associated with DNA abnormalities in the babies’ blood. This type of permanent genetic alteration has been linked to increased risk of cancer in children and adults. Also, PAH-induced DNA damage in the babies, in conjunction with exposure to secondhand tobacco smoke, was associated with significantly lower weight and smaller head circumference at birth—both signs of potential future developmental and learning problems.

The pesticide exposures of mothers and children in these urban communities occurred mainly due to insect and rodent infestations in poor-quality housing. *In utero* exposure to two organophosphate pesticides, chlorpyrifos (then the most widely used pesticide in New York City) and diazinon, resulted in an average birth weight reduction of 6.6 ounces, says Robin Whyatt, an associate professor of clinical environmental health sciences and co-deputy director of the Columbia center.

In 2000, however, the EPA announced that chlorpyrifos and diazinon would both be banned as household pesticides, and “the levels of pesticides in air during pregnancy and in the blood of both mothers and newborns were sharply reduced,” says Perera. By the time samples were taken in the spring of 2001, researchers no longer saw an association between organophosphate exposure and low birth weight.

Columbia researchers are also involved in a number of other studies, including an intervention project to reduce toxic pesticide use in public housing, says Perera. Residents are taught integrated pest management techniques, including removing pest food sources, sealing cracks and crevices, and using low-toxicity pesticides such as baits, gels, and boric acid. The families involved are also given lidded trash containers, pest-proof food containers, trash bags, and cleaning supplies.

## Mount Sinai School of Medicine

At the Mount Sinai center, scientists are also using a pregnancy cohort of about 400 New York women of different ethnicities who gave birth at Mount Sinai Hospital from 1998 to 2003. During this period, the study focused on pesticide exposure and how genetic variations in the paraoxonase 1 (PON1) enzyme—which detoxifies organophosphate pesticides in the body—modify response to pesticides.

Maternal blood samples were taken during the third trimester, and PON1 activity was assessed. In the March 2004 issue of *EHP*, the Mount Sinai researchers reported finding that infants exposed to chlorpyrifos *in utero* were born with smaller head circumferences, but only if their mothers also had low levels of PON1 activity, says center scientist James Wetmur, a professor of microbiology and human genetics.

In 2003, these studies of development and genetic susceptibility moved away from organophosphate pesticides, largely because levels of these chemicals dropped after the EPA ban on residential use, says Mary Wolff, director of the Mount Sinai center and a professor of community and preventive medicine and oncological sciences. Researchers at Mount Sinai are now focusing on *in utero* exposures to endocrine-disrupting chemicals often found in plastics such as phthalates and phenols such as bisphenol A.

The center preserves biologic samples from all cohort members for future studies, Wolff says, so researchers will be able to analyze phthalate and phenol levels in maternal prenatal urine samples and correlate these levels with birth outcomes and with subsequent growth and neurodevelopment. Wetmur and his team are also switching gears to search for enzymes that metabolize phthalates and phenols, as well as for genetic variation in these enzymes that might affect birth or growth outcomes.

In a separate study in East Harlem, center researchers have found that integrated pest management is effective at controlling cockroaches. In addition to reducing or eliminating exposure to toxic pesticides, the long-term cost of this method—including building repairs—is lower than standard chemical-based pest control, making it available to lower-income residents. According to a report in the October 2003 *EHP*, “The costs of adopting building-wide integrated pest management in a typical East Harlem apartment building were calculated to be $46–69 per unit in the first year (including repairs) and $24 per unit per year in subsequent years,” compared to $24–46 per unit per year, not including repairs, for traditional chemical-based control. In coming years, this intervention project will next look at how the built environment affects exposures to endocrine-disrupting chemicals.

Another study to find evidence of health effects of PCBs showed that early-life exposure to these chemicals in animals can affect neuroendocrine development. Led by neuroendocrinologist Andrea Gore, then at Mount Sinai and now at the University of Texas at Austin, researchers discovered that these chemicals directly influence brain cells called gonadotropin-releasing hormone (GnRH) neurons. These neurons control reproduction in all vertebrates, and disruption in their growth or activity can lead to fertility problems, Gore says. In the October 2002 issue of the *Journal of Neuroendocrinology*, Gore and colleagues reported that a more estrogenic PCB mixture, Aroclor 1221, stimulated GnRH expression, while the less estrogenic Aroclor 1254 had both stimulatory and inhibitory effects, depending on the transcript measured.

## University of California, Berkeley

As at Columbia and Mount Sinai, researchers at UC Berkeley are conducting a prospective cohort study of pregnant women and their children. Most women and children enrolled in this study—about 600 pairs total—come from low-income Mexican immigrant farmworker families who live in California’s Salinas Valley.

The first goal of the study has been to understand levels and routes of exposure to pesticides and other environmental contaminants among pregnant women and children, says Brenda Eskenazi, the center director and a professor of maternal and child health and epidemiology. Researchers have collected samples of urine, breast milk, blood, and house dust. They are determining the relationship of urinary pesticide metabolites in pregnant women and children with levels of pesticides in house dust, parental occupation, and nearby agricultural pesticide applications. They are also videotaping young children to identify behaviors that may expose them to environmental chemicals.

They’ve found that pregnant women in their cohort show abnormally high urinary levels of organophosphate pesticide metabolites, with about 15% of them likely exceeding the maximum cumulative exposure levels advised by the EPA. Organophosphate metabolites were higher in 6-month-old babies if the children lived with an agricultural worker. These metabolite levels were also significantly correlated with season, with urine collected in the summer showing the highest concentrations of pesticides. Levels of pesticide metabolites in urine rose as the children passed 6 months, likely because their activity levels—especially hand-to-mouth behavior—increased as they grew older.

A second goal of the study is to examine the health effects of these pesticide exposures in the children of exposed mothers. As in the Columbia and Mount Sinai studies, children in this cohort will be followed through at least age 7 to determine whether prenatal and childhood exposures have altered their cognitive development, growth, or respiratory health. UC Berkeley scientists have already found that high maternal organophosphate exposure during pregnancy correlated with shorter gestation duration, but no associations were found between organophosphate exposure and infant birth weight, length, or head circumference. A UC Berkeley center study published in the March 2005 issue of *NeuroToxicology* showed that newborns whose mothers had high levels of pesticide metabolites during pregnancy were more likely than other babies to have abnormal reflex functioning soon after birth.

The UC Berkeley center’s projects also include a randomized intervention study to see what types of preventive measures best discourage pesticide transmission from farmworkers to their children, Eskenazi says. Other projects include examining pesticide levels in amniotic fluid and breast milk, monitoring ambient pollen and mold levels, and studying mechanisms of pesticide and allergen effects on neural and immune functions.

## University of Washington

The UW center also is measuring the extent of pesticide exposure in agricultural communities. Building upon previous UW center research in the Yakima Valley, center researchers have found that children of orchard workers can be exposed to pesticides that are transported on the clothing, boots, and skin of their farmworker parents, says center director and professor of environmental health Elaine Faustman. These studies also linked children’s exposure with specific agricultural crops, which will be detailed in upcoming unpublished papers. Such findings will allow the UW center to intervene more effectively in preventing the occupational take-home pathway for pesticide exposure in children.

UW scientists have also developed a laser-based method that allows them to monitor pesticide spray drift in real time. They’ve shown that pesticides can volatilize unexpectedly in certain conditions, especially in extreme heat—so even though time has elapsed since a crop spraying, it still may not be safe for children to go near the fields. These results should influence EPA recommendations for safety near agricultural fields after pesticide application, Faustman says.

A major part of UW research has focused on genetic susceptibility to the neurotoxic effects of organophosphate pesticides. Using data gathered by the UC Berkeley center, UW researchers have shown that people with certain forms of the PON1 gene break down chlorpyrifos more efficiently than people with different forms, although all forms detoxify diazinon at the same rate.

However, knowing which genetic variant a person has does not tell you what level of PON1 is present in the blood, says UW research professor of medical genetics Clem Furlong. Knowing the activity levels of the enzyme is important in determining how well a person will metabolize organophosphates and the potential for health impacts from organophosphate pesticide exposure.

“I think it’s extremely important to emphasize that, because epidemiologists continue to try and estimate risk only by doing genotype,” Furlong says. “You really need to look at the functional status of individuals.” It takes nearly a year for infants to begin making the amount of PON1 they will have as adults, and this may lead to increased vulnerability to exposure during this time, says Furlong. Maternal PON1 can provide some protection *in utero*, but “if you have a mother with extremely low PON1 levels, this is a serious concern—there’s no ability of that fetus to protect itself,” he says.

In animal studies, UW researchers have examined the mechanisms through which pesticides cause neurotoxicity. They’ve found that different pesticides can have very different influences on cell proliferation, differentiation, and death during brain development, and all of these effects are dependent upon dose and time of exposure during development. For example, in the March 2004 issue of *Toxicological Sciences* the team reported that chlorpyrifos induced apoptosis in primary cortical neurons cultured from embryonic and newborn rats. Currently, says Faustman, center researchers are expanding studies in mice to see how the combination of exposure and genetic susceptibility affects behaviors in the animals.

## Where to Go From Here

The research coming out of these children’s centers over the past seven years has revealed that there are still far more unknowns than knowns, says Nina Holland, an adjunct professor of environmental health sciences at Berkeley and member of the UC Berkeley center. There are also discrepancies between some of the findings emerging from different centers. For example, the Columbia center’s report in the July 2004 issue of *EHP* that *in utero* exposure to chlorpyrifos or diazinon resulted in an average birth weight reduction of 6.6 ounces contrasted with a UC Berkeley study in the same issue, which found no adverse relationship between fetal growth and any measure of *in utero* organophosphate pesticide exposure (in fact, that team found increases in body length and head circumference associated with some exposure measures). But the overall finding, Holland says, is that “we have to pay much more attention to potential effects of pesticides on very young children.”

Center researchers are translating their experimental results into interventions, educational materials, community forums, press releases, and newsletters that can be used by parents, health care providers, and policy makers to improve the environmental health of local children. For example, the Columbia center has established a community educational campaign called “Healthy Home, Healthy Child.” Through this program, they have surveyed parents and caretakers of children in Harlem, Washington Heights, and the South Bronx to determine what these people are most concerned about. Then they’ve compiled tip sheets on topics such as air pollution, tobacco smoke, nutrition, pesticides, and lead poisoning, and they’ve distributed these on the street and at community health fairs and public forums. Center researchers have also trained staff at community centers to deliver health workshops to many different types of local groups, such as parent–teacher associations, churches, after-school programs, and foster care agencies. They also send summaries of their findings—in English and Spanish—to all the mothers involved in these cohort studies.

One important focus for the future is the National Children’s Study, according to Nsedu Obot Witherspoon, who is executive director of the Children’s Environmental Health Network. Slated to begin enrolling in the fall of 2007, the proposed study will follow 100,000 children from preconception or early pregnancy through adulthood, examining the effects of many different environmental exposures on various health outcomes. Leaders of the study include the NIEHS, the National Institute of Child Health and Human Development, the Centers for Disease Control and Prevention, and the U.S. EPA.

Researchers from all of the children’s centers and from the National Children’s Study should be able “to work hand in hand and will provide a wealth of information we would have otherwise never had,” Obot Witherspoon says. “It’s going to be phenomenal.”

## Figures and Tables

**Figure:**
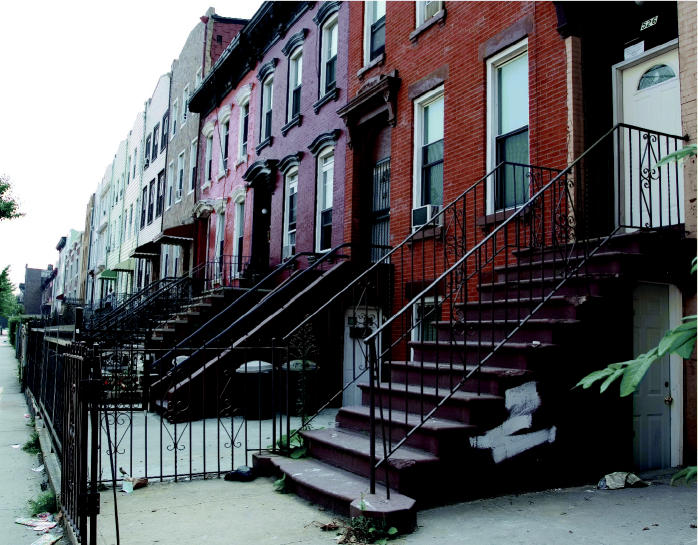
Home is where the exposure is. A myriad of exposures in low-income urban housing—including vehicle exhaust and pesticides used in homes—contribute to conditions ranging from cancer to low birth weight.

**Figure:**
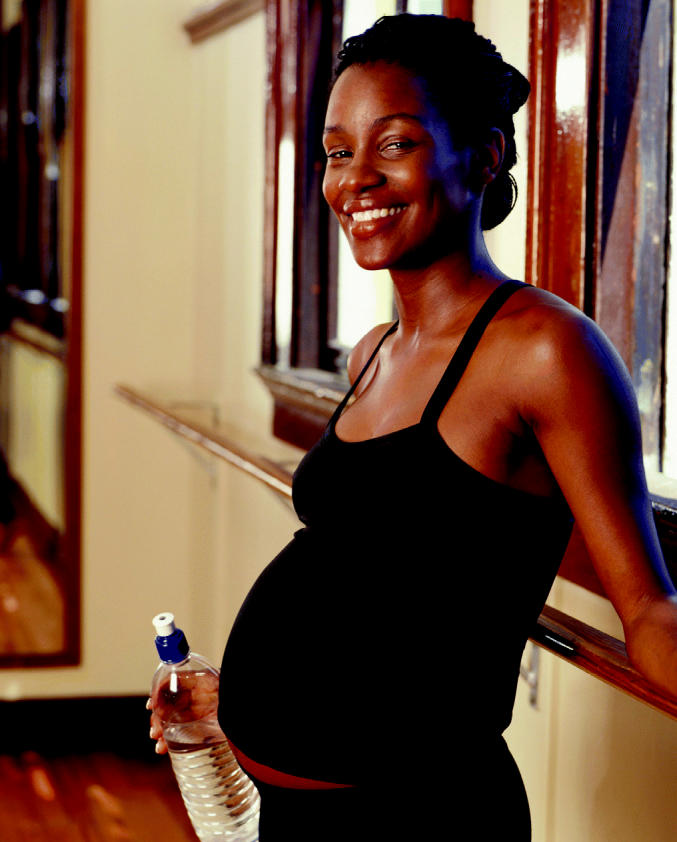
Mothers, babies, and chemicals. Researchers are studying whether variations in the enzymes that metabolize the phthalates found in some plastic bottles correlate with later birth and growth outcomes.

**Figure:**
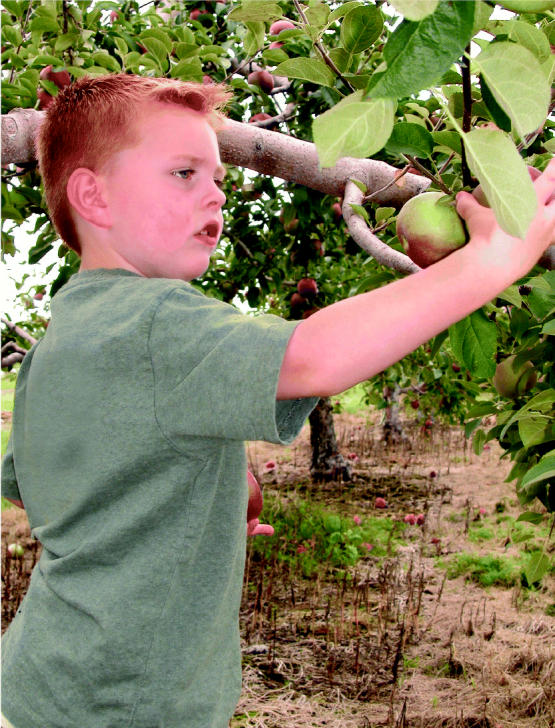
More than one way in. Through pathways both expected and surprising, children of farmworkers have higher pesticide exposure than the general population.

**Figure:**
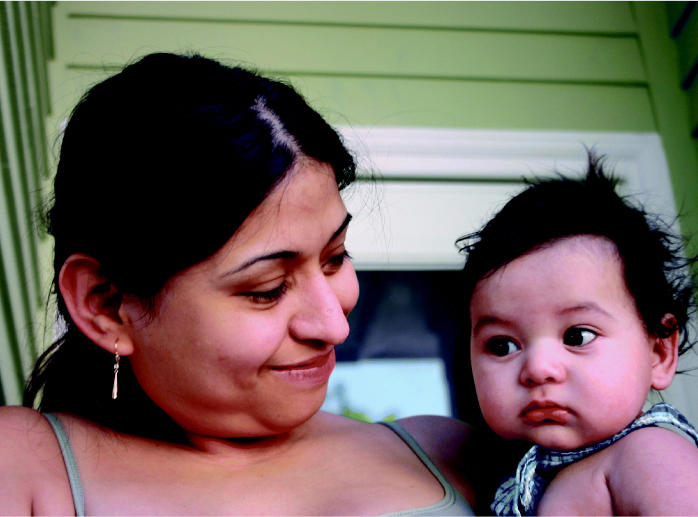
Waiting for the future. A study of Mexican immigrant farmworkers will follow children through at least age 7 years to monitor possible effects of prenatal and childhood exposure to pesticides.

